# An optical Fourier transform coprocessor with direct phase determination

**DOI:** 10.1038/s41598-017-13733-1

**Published:** 2017-10-20

**Authors:** Alexander J. Macfaden, George S. D. Gordon, Timothy D. Wilkinson

**Affiliations:** 1 Centre of Molecular Materials for Photonics and Electronics, Department of Engineering, University of Cambridge, 9 JJ Thomson Avenue, Cambridge, CB3 0FA UK; 2 Optalysys Ltd., Flemming Court, Whistler Drive, Glasshoughton, WF10 5HW UK

## Abstract

The Fourier transform is a ubiquitous mathematical operation which arises naturally in optics. We propose and demonstrate a practical method to optically evaluate a complex-to-complex discrete Fourier transform. By implementing the Fourier transform optically we can overcome the limiting *O(nlogn)* complexity of fast Fourier transform algorithms. Efficiently extracting the phase from the well-known optical Fourier transform is challenging. By appropriately decomposing the input and exploiting symmetries of the Fourier transform we are able to determine the phase directly from straightforward intensity measurements, creating an optical Fourier transform with *O(n)* apparent complexity. Performing larger optical Fourier transforms requires higher resolution spatial light modulators, but the execution time remains unchanged. This method could unlock the potential of the optical Fourier transform to permit 2D complex-to-complex discrete Fourier transforms with a performance that is currently untenable, with applications across information processing and computational physics.

## Introduction

Historically, optical information processing (OIP) has manifested itself in many imaginative–and some successful–ways: from image processing^1–3^ and pattern matching^4^, to numerical equation solving^5^ and even to implementing a general purpose digital computer^6^. Despite this rich and prodigious history, OIP has often failed to compete with the formidable progress of digital electronic computers^7^. However, it still potentially offers significant advantages over electronic methods when used appropriately^8–10^. Specifically, we consider the natural application of the Optical Fourier Transform (OFT): replacing the Discrete Fourier Transform (DFT), as normally implemented by a Fast Fourier Transform (FFT) algorithm, with a dedicated optical coprocessor.

The Fourier transform at the core of coherent optics is straightforwardly implemented with a classic *2f* system^11^. However, using this phenomenon to evaluate a complex-to-complex Fourier transform requires full complex control of the light at the input spatial light modulator (SLM), and measurement of the complex amplitude across the focal plane at the output. While a camera sensor can straightforwardly measure the magnitude, determining the phase is a venerable problem. Historical approaches include: iterative algorithms^12^ which are too slow for a Fourier coprocessor; direct methods based on including a reference in the input plane^13^; and of course interferometry.

If we could perform the Fourier transform optically, we would escape the ${\mathscr{O}}(n\,\mathrm{log}\,n)$ complexity of the FFT^14^. Moreover, as performing large FFTs is a memory-bandwidth limited operation, the real obstacle to performing arbitrarily large DFTs is that it becomes difficult to implement the FFT algorithm across many parallel processors. Beyond a certain point, ${\mathscr{O}}(nlogn)$ scaling is hard to achieve^15^. In contrast, when evaluating larger DFTs optically–provided we have sufficient input and output resolution–our ‘computational resource’ is free space and the wall-clock time is unchanged for larger resolutions. Thus, provided we can marshal optical hardware with a sufficient number of pixels, the OFT exhibits a very favourable scaling regime.

In this paper, we first present the method used to directly determine the phase of the Fourier transform of a complex function from intensity measurements. Then, we provide an experimental demonstration. Finally, we discuss how this method overcomes the limitations of the FFT implemented on a digital computer and the different set of trade-offs it introduces.

## Method

**Figure 1:**
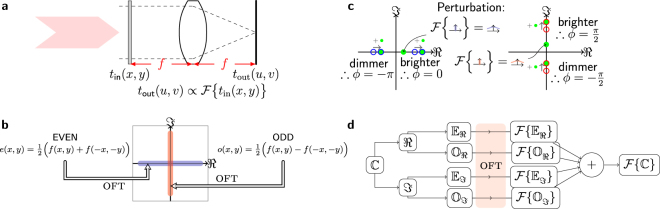
(**a**) The classic *2f* architecture, where a simple lens renders the complex Fourier transform of the amplitude distribution at one focal plane, ${t}_{in}$, at the other, ${t}_{out}$. (**b**) The Fourier transform of an even function is constrained to be a purely real function. All points of this function lie on the horizontal real axis of the Argand plane. Similarly, the Fourier transform of an odd function is imaginary, and this function is constrained to the imaginary vertical axis of the Argand plane. A perturbative principle will allow us to distinguish the binary phase values by the interaction of the Fourier plane with a field of known phase. (**c**) The perturbative principle by which the constrained phase is determined. A zero-frequency perturbation applied at the input results in a broad perturbation in the Fourier plane which interacts with the points in the Fourier plane. Depending on whether the perturbation is *in-phase* or *anti-phase* with the spot, it gets brighter or dimmer respectively. (**d**) A general complex OFT can be found by decomposing it into even and odd components, finding the OFTs, and then summing the results.

It is well known that the Fourier transform of a coherent optical field at the front focal plane of a lens is rendered at the back focal plane, as shown in Fig. (1a). A simple $2f$ optical system uses a pixelated SLM at the front focal plane to encode information onto an optical field, and a camera at the back focal plane to record the optical power distribution. In order to make use of this to evaluate optical Fourier transforms, a challenge is to determine the optical phase at the output with an efficient algorithm using only straightforward intensity measurements.

We propose a method which exploits the following symmetries of the Fourier transform, ${\mathcal F}$ ^16,17^:

1 $$T(u,v)={\mathscr{F}}\{t(x,y)\}=\{\begin{array}{c}{\rm{r}}{\rm{e}}{\rm{a}}{\rm{l}},{\rm{e}}{\rm{v}}{\rm{e}}{\rm{n}}\,{\rm{f}}{\rm{o}}{\rm{r}}\,t\,{\rm{r}}{\rm{e}}{\rm{a}}{\rm{l}},{\rm{e}}{\rm{v}}{\rm{e}}{\rm{n}},\qquad \\ {\rm{i}}{\rm{m}}{\rm{a}}{\rm{g}}{\rm{i}}{\rm{n}}{\rm{a}}{\rm{r}}{\rm{y}},{\rm{o}}{\rm{d}}{\rm{d}}\,{\rm{f}}{\rm{o}}{\rm{r}}\,t\,{\rm{r}}{\rm{e}}{\rm{a}}{\rm{l}},{\rm{o}}{\rm{d}}{\rm{d}}.\end{array}$$

Thus, the optical Fourier transform of an even function has phases constrained to ${\varphi }=\mathrm{\{0,}\,\pi \}$ and an odd function to ${\varphi }=\{-\tfrac{\pi }{2},\tfrac{\pi }{2}\}$, as shown in Fig. (1b). The symmetry constraint of displaying an even or odd function sacrifices half of the spatial bandwidth of the system, but has the benefit of highly constraining the phase.

To perform a complex-to-complex Fourier transform and take advantage of these symmetries we must decompose a general complex input function $C$ into two real functions such that $C={R}_{1}+i{R}_{2}$, which are decomposed again into even and odd components such that ${R}_{{\rm{k}}}={E}_{{\rm{k}}}+{O}_{{\rm{k}}}$ $(k=\mathrm{1,}\,\mathrm{2)}$. By the linearity of the Fourier transform, the transform of $C$ is the sum of the transforms of these four constituent functions. We evaluate these OFTs independently, before recombining as shown in Fig. (1d).

To determine which of the constrained phases (${\varphi }=\mathrm{\{0,}\,\pi \}$ and ${\varphi }=\{-\tfrac{\pi }{2},\tfrac{\pi }{2}\}$ for the even and odd case respectively) is present at each point in the constituent OFTs we apply a small zero-frequency perturbation at the input, as shown in Fig. (1c). This is implemented by increasing the amplitude transmitted by the central pixel, resulting in a broad perturbation of known phase in the output which interacts with the OFT. If the perturbation is in-phase with the OFT, then the output point gets brighter; if the perturbation is anti-phase with the OFT, the points gets dimmer. Essentially, we are using common path interferometry between the OFT and the perturbation (both of which originate at the SLM) to allow direct determination of the phase simply from the sign of the difference in intensity. This is superior to other interferometric approaches in that both beams come from the SLM so there is little noise. Also, because we are only trying to determine the sign of the change the requirements on precision are relaxed.

The ideal perturbation would be only at zero frequency, as this would result in a constant perturbation applied across the Fourier plane. However, the finite pixel size of the SLM precludes an ideal DC perturbation. There are two ways of considering this situation. Firstly, the envelope function due to the pixel shape can be considered explicitly and applied appropriately to both the function and the perturbation. Alternatively, it can be acknowledged that the effect of the envelope function is to apply a multiplicative complex function to both the input and the perturbation. This effect is effectively ‘cancelled out’™ as we are only looking for the relative phase between these two functions. Hence, the more sophisticated analysis is in fact not required.

**Figure 2:**
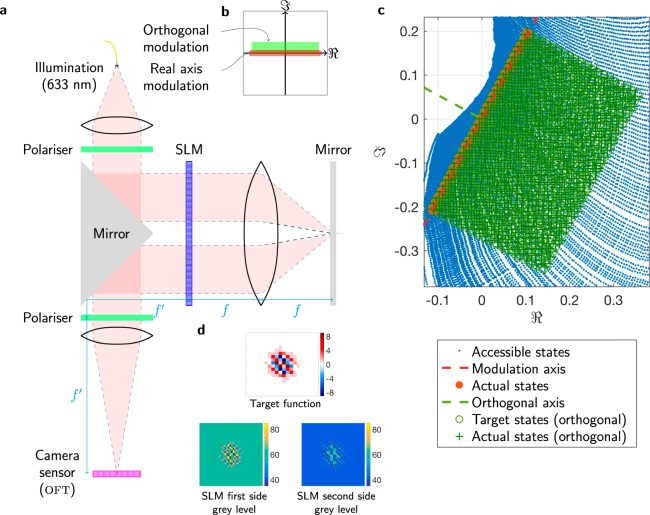
(**a**) The two-pass SLM system used to achieve the appropriate optical modulation, followed by a lens which renders the optical Fourier transform on the camera sensor ($f={f}^{\text{'}}=100$ mm). (**b**) The required optical modulation: real axis modulation to display the functions, with orthogonal modulation across states to implement the imaginary perturbation required by the odd function. (**c**) The available modulation states implemented by the two-pass system, showing the real-axis and orthogonal modulation (to within a rotation representing a global phase). (**d**) An example of a target real-axis function (top), and how this is implemented by showing appropriate complementary images on the first side and second side of the SLM (bottom). Note there is a flip due to reflection by the mirror.

Since the proposed method requires the ability to display even and odd functions, continuous-amplitude binary-phase (real axis of the complex plane) optical modulation is necessary. Furthermore, in order for the perturbation to interact strongly with the OFT it should be exactly in-phase or anti-phase to the OFT. The real OFT of the even function requires a real perturbation; the imaginary OFT of the odd constituent function requires an imaginary perturbation. Our perturbation function is always an even function as it consists of a single central pixel straddling $x=0$. Hence the complex modulation capability required is real axis plus an extension orthogonally to allow for the imaginary perturbation, as summarised on the Argand diagram in Fig. (2).

### Apparatus and implementation

This modulation capability is implemented using the system in Fig. (2a). (See Macfaden *et al*.^18^ for a full description of this system.) Coherent laser light (632.8 nm from a HeNe laser) passes sequentially through each side of an 8-bit twisted nematic SLM (Holoeye LC 2012). The first side is imaged onto the second side using a folded $4f$ optical relay. The relatively large pixel pitch (36  *μ*m) and deadspace (58% fill factor) of this SLM mean the relay exhibits minimal optical crosstalk. The combined action of the two sides of the SLM act as the input to the system. An *in situ* characterisation of the SLM’s modulation at each grey level (using a Jones matrix formalism) is used to design an appropriate polariser configuration. Combinations of levels on each side of the SLM are then used to implement a prescribed optical modulation. A sub-set of the ${{\mathrm{(2}}^{8})}^{2}$ accessible states in an optimised polariser configuration provide the required modulation scheme, as shown in Fig. (2c) (to within a rotation, which represents an arbitrary global phase delay).

In order to increase brightness–albeit while reducing the effective resolution–each point on the input function is represented by four pixels on the SLM to increase brightness, and a Nyquist-frequency chequerboard is used to carry the OFT away from the zero-order point to avoid interference. This effect can be seen in the SLM drive levels shown in Fig. (2d).

The Fourier transforming lens has a focal length of 100 mm. The Fourier plane is captured by a Ximea MQ0013MG-E2 monochrome board-level camera sensor, operating at 8-bit precison. The camera pixel pitch is 5.5 *μ*m. The respective resolution of the SLM and camera is $768\times 1024$ and $1280\times 1024$. Neither of these components are used to their full potential due to optical limitations of the system. A higher bandwidth system would require a custom optical design.

Note that this method can also be applied to other Fourier transform cases, such as real-to-complex and complex-to-real. In the former case, only one branch of the operations shown in Fig. (1d) is used. In the latter case–provided the symmetry of the input complex function ensures that the output is real (i.e. $f(x,y)=f(-x,-y)$)–the complex function is displayed directly, and the phases to be determined are either $0$ or $\pi$. This requires full-complex optical modulation ability, rather than the real-axis modulation demonstrated in Fig. (2).

### Data Availability

Open access data can be found at https://doi.org/10.17863/CAM.13408.

## Results

### Phase determination of the OFT of an even function

**Figure 3:**
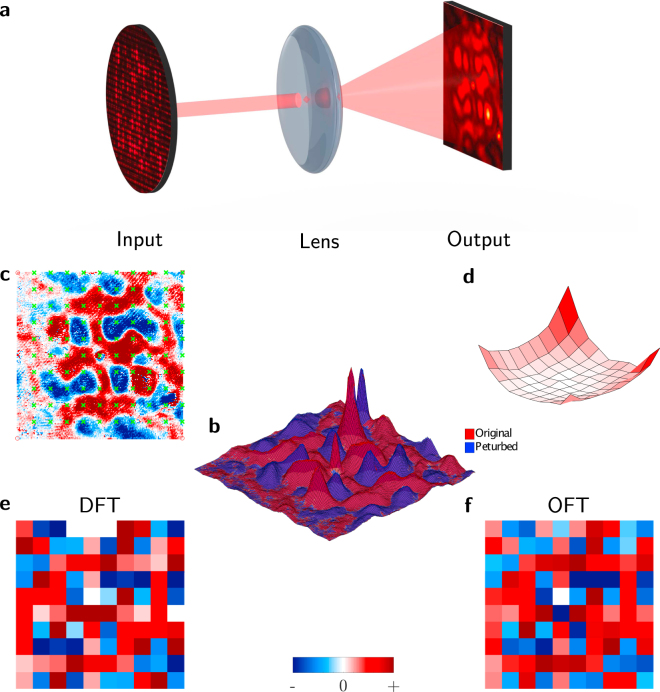
(**a**) The Fourier transform of the input (even) function is rendered on a camera sensor by a lens. The perturbation is indicated by the visible beam. (**b**) Surfaces corresponding to the original and perturbed function intensities at the focal plane, showing the change under perturbation. (**c**) The difference between the original and perturbed function, and appropriate sampling points to obtain the DFT. (**d**) The envelope correction function required to compensate for the pixel apodization. For comparison, (**e**) the computed DFT for the presented function, and (**f**) the OFT.

An experimental demonstration of the determination of the phase of the complex optical Fourier transform of an even function is shown in Fig. (3a). The even function is encoded onto the optical field using the system shown in Fig. (2). A camera sensor measures the resulting power spectrum of the Fourier transform. The magnitude of the amplitude is found by taking the square root of the intensity. As we displayed an even function, the symmetry properties of the Fourier transform constrain the phase values to ${\varphi }=\mathrm{\{0,}\,\pi \}$ (i.e. the function is real). These two phases are distinguished by applying a perturbation. Specifically, the amplitude of the central pixels on the SLM is increased, and the new OFT recorded. The two resulting OFT power-spectra (unperturbed and perturbed) are shown as surfaces in Fig. (3b). The effect of the perturbation is to increase or decrease the intensity at different parts of the OFT, as shown in Fig. (3c). The difference between these two images encodes the phase.

The optical system is performing a true continuous Fourier transform (albeit of a sampled input), while we are interested in performing the discrete Fourier transform. Thus we need to sample the OFT appropriately. An ideal sensor array would implement Dirac comb sampling with zero-size pixels. However, this is clearly not practical. We tend towards this case by over-sampling the OFT with the camera sensor and selecting a sparse subset of the sensor pixels to represent the DFT values. These are shown as green crosses overlaid with the difference due to the perturbation in Fig. (3c). In a competitive implementation of this technology, a custom sparse sensor array should be implemented.

Furthermore, in the DFT the input function is sampled with a Dirac comb rather than the square SLM pixels used here. Hence, our output will be modulated by a sinc envelope due to the pixel shape. This envelope is measured experimentally, as the effective pixel-shape (which leads to apodization of the Fourier transform) in the two-pass system depends on alignment. The appropriate correction function is shown in Fig. (3d). The digitally computed DFT and optically computed OFT are shown in Fig. (3e) and Fig. (3f) respectively. There is good agreement between the optical measurement and the computed values. Note that these results are unprocessed and do not include any error-reduction techniques.

### Visualisation of a heat-equation simulation

We now turn to results from a ‘toy problem’: using the optical Fourier transform to visualise the evolution of a system modelled by the heat equation,

2 $$\frac{\partial q}{\partial t}=\alpha {\nabla }^{2}q,$$

where $q(x,y,t)$ is the evolving 2D temperature profile and $\alpha$ is a positive constant. A classic method to numerically simulate such a system is to exploit the fact that in the Fourier domain the heat equation becomes

3 $$\frac{\partial Q}{\partial t}=\alpha ({\mathrm{(2}\pi iu)}^{2}+{\mathrm{(2}\pi iv)}^{2})Q,$$

where $Q(u,v,t)$ is the Fourier domain representation of the heat distribution. A simple implicit finite difference method can be used to model the system evolution as

4 $${Q}_{t+1}={Q}_{t}(1+\delta t\alpha ({\mathrm{(2}\pi iu)}^{2}+{\mathrm{(2}\pi iv)}^{2})),$$

where $\delta t$ is a timestep. By evaluating in Fourier space, instead of calculating a computationally costly differential each timestep we evaluate a cheap multiplication instead. However, to visualise the progress of this simulation we need to use the FFT, which would become computationally costly at high resolutions. Moreover, if we were considering a non-linear differential equation we would be obliged to use a FFT each timestep in order to evaluate the non-linear terms. Hence, it is compelling to consider using the optical Fourier transform to visualise the progress of such a simulation. This requires performing a set of complex to real (as temperature is a real quantity) Fourier transforms. We do this using the full method shown in Fig. (1d), but where the output happens to be real.

**Figure 4:**
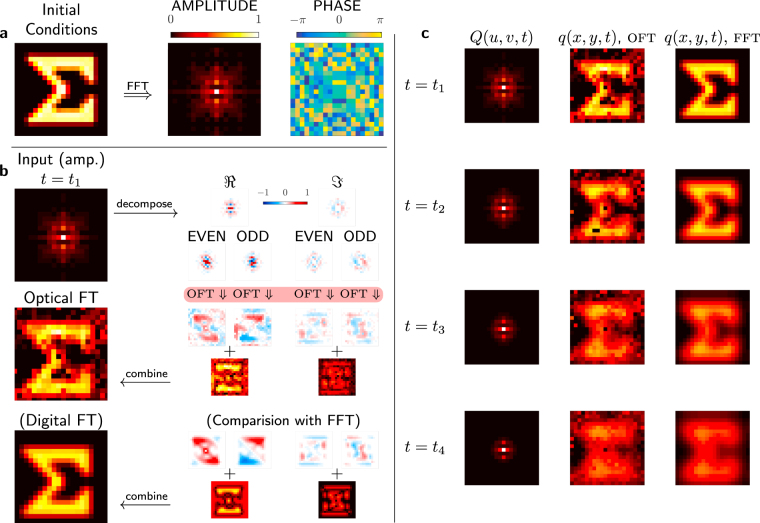
Using the OFT to visualise the progress of a Fourier domain heat equation simulation. Note all equivalent quantities are evaluated with the same normalised units. (**a**) The initial conditions for the simulation and the amplitude and phase respectively of the corresponding Fourier domain representation $Q(u,v,t=\mathrm{0)}$. (**b**) Applying our method to determine the heat distribution at a subsequent time ${t}_{1}$. The complex input function (phase not shown) is decomposed into real and imaginary, then even and odd components. These components all have binary phase. The OFT is then performed using the $2f$ system, and the phase determined using the response of this function to the perturbation. These components can then be added together to form first the Fourier transform of the real and imaginary components, and then the Fourier transform of the entire function (which happens to be real). The Fourier representations (inputs) have been DC balanced, and the central order DC term of the direct (output) representation must be ignored due to system imperfections. **(c)** The temperature distribution evaluated optically in the same way at subsequent times, compared with the same calculated by a DFT on a computer. (See Supplementary Video 1 and 2 for a raw and temporally smoothed animation, respectively, of more cooling stages).

A low-resolution demonstrating of using the OFT to visualise the progress of one such simulation is shown in Fig. (4). The initial conditions for the simulation are shown in Fig. (4a). An example of the stages of a complete complex-to-complex OFT is shown schematically in Fig. (4b). The function is decomposed into its even and odd components, which are displayed on the SLM and the power spectrum obtained. The phase of these optical Fourier transforms is constrained by the symmetry of the functions. A perturbation is applied to determine the phase. The discrete Fourier transform, which happens to be real, is then reconstituted from these components. Subsequent results at different timesteps are shown in Fig. (4c). It is clear that there is in general a good recovery of the overall result and the phase determination method is working well. The error observed is the result of noise during acquisition and imperfect optical modulation.

## Discussion

### Utility of an optical Fourier transform coprocessor

Digital electronic computers are a prodigiously successful technology, and the fast Fourier transform is a ubiquitous and highly optimised algorithm^19^. Nonetheless, there is potentially utility in an optical Fourier transform coprocessor. Performing extremely high resolution 2D Fourier transforms can often represent a computational bottleneck. An optical Fourier transform coprocessor is potentially a compelling solution to this problem as it provides a different scaling paradigm. As well as offering improved performance, coherent optical processing offers the potential for significant improvements in power efficiency as only the input and output components consume power. However, in order for such a system to prove useful, a sufficiently large number of pixels must be marshalled.

Moreover, an analogue optical processor of this nature cannot rival the precision and accuracy offered by a digital electronic computer. The maximal accuracy which could be obtained in a single calculation is likely significantly less than 1 byte (8 bits). Higher precision transforms would require computing lower precision transforms and combining them, exploiting the linearity of the Fourier transform. However, this would require high confidence in the given accuracy, in particular of the transforms representing the more significant bits. In order to compete, the advantage offered in terms of resolution and execution time must be overwhelming compared to the digital alternative.

Critically, all of the computational steps in this algorithm–decomposing the function, applying the perturbation, observing the change, and combining the components–are acting on individual pixels independently (except for the decomposition step, which acts on pairs of opposing pixels at $(\pm x,\pm y)$), and hence are easy to parallelise. Meanwhile, the difficult-to-parallelise, highly connected problem of the Fourier transform (every point in the output depending on every point in the input) is implemented optically. In the OFT these cascaded addition operations are instead achieved by the linear superposition of wavefronts giving an apparent ${\mathscr{O}}\mathrm{(1)}$ behaviour. The decomposition and reconstitution steps scale linearly meaning that overall the algorithm will scale as ${\mathscr{O}}(n)$.

### Technical challenges

A particular problem is that cameras measure optical intensity rather than the relevant ${\rm{amplitude}}=\sqrt{{\rm{intensity}}}$. Consequently many of the amplitude levels are towards the bottom of the intensity scale. A non-linear intensity response in an appropriately-corrected camera–essentially a specific hardware gamma correction–could reduce this. Furthermore, achieving accurate high-resolution Fourier transforms places high demands on the optical design required to achieve a well-corrected distortion- and aberration-free Fourier transform.

As with all analogue processing systems one must contend with the fact that precision is limited by inherent physical noise. While higher quality components can be used to reduce its effect, in a coherent system such as this optical speckle is a significant (and inevitable) source of noise^20^. This can be reduced through diverse measurements, for example by taking the OFT of the same function rotated, translated, and scaled on the SLM and averaging the results. Precision can be increased by combining the linear FFTs across different levels. While this has an associated computational cost (${\mathscr{O}}(n)$), it is critically not as demanding as the highly connected problem of finding a Fourier transform and, unlike the FFT, it can be straightforwardly parallelised. Furthermore, for a given precision input, higher precision computation happens naturally in the analogue optical domain.

The requirements on optical design in order to produce a well-corrected optical relay and a distortion-free optical Fourier transform are within the realms of conventional optical design^21^. However, as higher performance, high resolution, liquid-crystal on silicon (LCOS) devices are used, these tend to come with smaller pixels and the optical design issues become more challenging^22,23^. In particular, modern LCOS devices often have little inter-pixel deadspace, meaning that there is significant cross-talk in an optical relay in a two-SLM system. Both this requirement of well-isolated pixels, and that of small-size camera pixels would potentially require custom hardware development.

### Performance and outlook

Assuming that a digital electronic system can be built to drive the electro-optics to their full capacity, the factors limiting system performance are the hardware resolution and system refresh time. Greater than 100 megapixel camera sensors are now available, and perhaps making use of either physically or virtually tiled SLMs input resolutions of this order could be achieved with commodity SLMs. The slowest part of the system is likely to be the multi-level liquid crystal SLM, operating at $\sim 100$ Hz. As a comparison, a $7680\times 4320$ (33 megapixel) complex-to-complex single-precision 2D FFT on a contemporary NVIDIA K80 takes 46.2 ms, which would equate to a parallel optical system operating at 22 Hz. Thus, it can be seen that competitive performance is already achievable without significant input and output transducer development, although achieving sufficient system bandwidth is a challenge. This could be facilitated by tiling camera and SLM panels, notwithstanding some significant engineering challenges. It should be noted that this bandwidth limitation is not unique to an optical coprocessor, but a general issue with coprocessors.

While this potential performance is compelling, it must be acknowledged that existing computational technologies are prodigious and rapidly advancing. It is challenging to be competitive within this context. However, this method of performing an optical Fourier transform does benefit from an attractive scaling regime compared to other potential implementations, arising from the fact that the Fourier transform itself is implemented naturally in free space. Hence, it could very well prove attractive at extremely high resolutions. However, developing a very high resolution system comes with significant engineering hurdles. Not only is providing sufficient input and output bandwidth challenging, the optical design is indeed very demanding. We would contend, though, that the critical advantage of this technology is the scaling regime which it exhibits. Only the input and output bandwidth need scaling, the intermediate computational substrate does not. The challenge of providing sufficient interconnection in, for example, an electronic or optical integrated circuit to perform extremely high resolution Fourier transforms is considerable.

Indeed, it is at exceptionally high resolutions that the benefits of this method become significant, rendering the trade-offs less relevant. It is conceivable to evaluate 2D Fourier transforms at a scale and rate untenable with other methods and platforms. For example, there are applications–for example, processing data from systems such as the Square Kilometre Array–where access to extremely high resolution rapid Fourier transforms would be notable. Given the almost ubiquitous use of the Fourier transform this would have significant implications across many fields.

## Electronic supplementary material

Supplementary Video 1

Supplementary Video 2
